# Effect of Different Processing Methods on Quality, Structure, Oxidative Properties and Water Distribution Properties of Fish Meat-Based Snacks

**DOI:** 10.3390/foods10102467

**Published:** 2021-10-15

**Authors:** Asad Nawaz, Enpeng Li, Ibrahim Khalifa, Noman Walayat, Jianhua Liu, Sana Irshad, Anam Zahra, Shakeel Ahmed, Mario Juan Simirgiotis, Mirian Pateiro, José M. Lorenzo

**Affiliations:** 1 Key Laboratory of Plant Functional Genomics of the Ministry of Education/Jiangsu Key Laboratory of Crop Genomics and Molecular Breeding, College of Agriculture, Yangzhou University, Yangzhou 225009, China; 007298@yzu.edu.cn; 2 Co-Innovation Center for Modern Production Technology of Grain Crops, Yangzhou University, Yangzhou 225009, China; 3 Food Technology Department, Faculty of Agriculture, Benha University, Moshtohor, Benha 13736, Egypt; Ibrahiem.khalifa@fagr.bu.edu.eg; 4 College of Food Science and Technology, Zhejiang University of Technology, Hangzhou 310014, China; nomanrai66@zjut.edu.cn (N.W.); Jhliu@zjut.edu.cn (J.L.); 5 School of Environmental Studies, China University of Geo Sciences, Wuhan 430074, China; sanairshad55@gmail.com; 6 Islamabad Campus, University Institute of Diet and Nutritional Sciences, University of Lahore, Islambad 45750, Pakistan; Anam.zahra@dnsc.uol.edu.pk; 7 Campus Isla Teja, Instituto de Farmacia, Facultad de Ciencias, Universidad Austral de Chile, Valdivia 5090000, Chile; Shakeel1177@uach.cl (S.A.); mario.simirgiotis@uach.cl (M.J.S.); 8 Centro Tecnológico de la Carne de Galicia, Avd. Galicia No. 4, Parque Tecnológico de Galicia, San Cibrao das Viñas, 32900 Ourense, Spain; mirianpateiro@ceteca.net (M.P.); jmlorenzo@ceteca.net (J.M.L.); 9 Área de Tecnología de los Alimentos, Facultad de Ciencias de Ourense, Universidad de Vigo, 32004 Ourense, Spain

**Keywords:** cooking methods, fish meat snacks, LF-NMR, SEM, protein oxidation

## Abstract

Snack foods are consumed around to globe due to their high nutrition, taste and versatility; however, the effects of various processing methods on quality, structure and oxidative properties are scare in the literature. This study aims to evaluate the effect of various processing methods (frying, baking and microwave cooking) on quality, structure, pasting, water distribution and protein oxidative properties of fish meat-based snacks. The results showed that the frying method induced a significantly (*p* < 0.05) higher expansion than baking and microwave methods. Texture in terms of hardness was attributed to the rapid loss of water from muscle fiber, which resulted in compact structure and the increased hardness in microwave cooking, whereas in frying, due to excessive expansion, the hardness decreased. The pasting properties were significantly higher in baking, indicating the sufficient swelling of starch granules, while low in microwave suggest the rapid heating, which degraded the starch molecules and disruption of hydrogen bonds as well as glycosidic linkage and weakening of granules integrity. The water movement assessed by Low Field Nuclear Magnetic Resonance (LF-NMR) showed that frying had less tight and immobilized water, whereas microwave and baking had high amounts of tight and immobilized water, attributing to the proper starch-protein interaction within matrix, which was also evidenced by scanning electron microscopy (SEM) analysis. The protein oxidation was significantly (*p* < 0.05) higher in frying compared to baking and microwave cooking. The findings suggest the endorsement of baking and microwave cooking for a quality, safe and healthy snacks.

## 1. Introduction

Snack foods are popular around the globe due to their balanced nutrition, highly bioavailable bioactive compounds, specific aroma and taste as well as cost effectiveness [1]. Traditionally, these are made from wheat flour and frying methods, which lead to some undesirable consequences, such as low nutrition, high oil content [2], allergens to gluten protein [3] and poor water-holding capacity [4]. The role of processing methods has always been neglected or less perceivable influence, but these are of high importance in terms of loss of nutrition, structure, safety and physiochemical properties [5], and most recently alternation at molecular levels [6]. Thus, there is need to find some other alternatives of wheat flour as well as to find some other processing methods, which will not only improve the nutrition, but also improve the physiochemical properties. Thus, studying various processing methods and their impact on structure, quality and oxidative properties is inevitable.

Processing methods have a critical role in the quality and structure of food especially the mode of heat transfer, such as conduction (transfer of heat via intervening matter), convection (heat transfer by fluid) and radiation (heat transfer through space) [7]. Heat disrupts the native conformation of polypeptides, which hold the molecules together, resulting the increase in thermal motion, loss of secondary and tertiary structure [6] and rupturing the intermolecular forces, e.g., electrostatic or non-polar interaction and disulfide bonds [8]. Further, once proteins are unfolded in response of thermal processing, aggregation of proteins occur and scramble the disulfide bonding, resulting in side chain modifications [9], which cross link with other polypeptides that are exposed to oxidation. In addition, processing methods have significant influence in terms of structural modification [6], textural changes [10], water distribution [11], microstructural changes [12] and even oxidative changes [13]. On the other hand, the heating of starch granules results in the loss of crystalline structure as well as gelatinization, which changes the structure and composition of starch granules. Thus, studying these structural and physio-chemical modifications in the starch–protein matrix is foreseeable for a safe and healthy product, especially for snack foods that are consumed after every meal.

Fish meat has recently drawn a considerable attention of food industrialist due to its highly bioavailable protein [14], essential amino acids [15] and the fact that it is suitable for all religions in terms of religious concepts [16]. Since fish meat production has increased in the world, its use in processing food has received immense attention and has been used in combination with various other snacks foods, such as cassava starch [3], wheat flour [17], fish bone [2], potato powder [5] dietary fiber [18] and most recently the oxidation of protein, which, owing to various cooking methods and its relationship with the physiochemical properties of the end product has received considerable attention.

There have been many studies in the literature that focus on the nutrition, quality or the use of fish meat in snacks. However, there is no such report that narrates the effect of processing methods on the structure, quality and physiochemical properties of fish meat snacks. Therefore, the key aim of this study was to assess the effect of different processing methods (frying, baking and microwave heating) on structure, quality, and physiochemical properties of fish meat-based snacks. Snacks based on wheat flour were prepared by the partial replacement of fish meat and cooked with various different processing methods. The physiochemical properties, such as expansion, water hydration capacity and texture, were analyzed in order to assess the effect of different processing methods. Moreover, pasting properties were determined in order to predict the gelling behavior in different processing methods. In addition, water distribution properties were assessed in all processing methods using low-field nuclear magnetic resonance analysis. The study will provide useful information for the best use of processing method and open new window for the feasibility of different methods.

## 2. Materials and Methods

### 2.1. Materials

Grass carp (*Ctenopharyngodon Idella*, 1.5 ± 0.2 kg) in a deceased form was purchased from local supermarket of Yangzhou, Jiangsu, China. The composition of fish was as follows: moisture 77.5%, protein 18.5%, ash 0.9% and fat 2.35%. Wheat flour with 12% protein, 1.5% fat and 0.42% ash was acquired from Jinsha River industry Co., Ltd., Chengdu, Sichuan, China. Salt and cooking oil (sunflower oil, Arwana brand) were purchased from local supermarket. Potato powder (moisture 13%, protein 7.9% and fat 1%) was purchased from Zhang Jiakou Co., Ltd., Zhangjiakou, Hubei, China. All other chemicals used in this study were of analytical grade and were acquired from Sigma-Aldrich, Inc. (Natick, MA, USA), and were used without further purification.

### 2.2. Preparation of Snacks

Snacks were prepared according to the formulation of our previous study [14] with slight modifications. Briefly, wheat flour (70 g), potato powder (20 g), minced fish meat (10 g), tricalcium phosphate 0.03% *w*/*w* and salt (1.5%, *w*/*w*) were added in a mixing bowl, while 22 mL of water (32% *w*/*w* of wheat flour) was added into it and kneaded manually for 4 min until a uniform dough was obtained. The kneaded dough was covered by polythene sheet and kept at room temperature for proper hydration. After this, hydrated dough was rolled by a noodle-making machine (Model: FKM-180, Yongkang Electrical appliances, Jinhua, Zhejiang, China) roller in order to make a uniform sheet with a thickness of 3 mm. This uniform sheet was cut into square pieces of 3.5 × 3.5 cm, which were further dried in hot air oven for 2 h at 45 °C. The dried snacks were subjected to various cooking methods: frying (165 °C for 35–45 s) by a thermostatically controlled electric fryer (PFE8, IME, Salzburg, Austria), baking (220 °C for 6 min in a baking oven) was done in thermostatically controlled oven (Model: HGB-20D, Rudong Jiahua Food Machinery, Co., Ltd., Nantong, Jiangsu, China) and microwave cooking (1000 W for 3 min for each side) using a microwave oven (NE-1037, Panasonic, Osaka, Japan). The prepared snacks were packed in high-density polyethylene bags for further analysis. For each cooking method, cooking was performed in triplicates and for each batch, 1 kg of snacks were prepared with three replicates (3 kg for each cooking method).

### 2.3. Expansion of Snacks

The expansion of snacks was measured following the methodology of Nawaz, Xiong [19] with modifications. This was measured before and after cooking. For each cooking method, six snacks from each treatment were marked with permanent ink marker, and thickness (before and after cooking) was measured from a center point using digital Vernier caliper, having a minimum resolution of 0.01 mm. All values reported in results are mean of six replicates and were calculated by following formula:

Expansion (mm) = Thickness before cooking − Thickness after cooking

### 2.4. Physicochemical Properties

Water hydration capacity (WHC) was calculated by following the study of Nawaz, Xiong [20]. All values reported in the results are mean values of triplicates. Color parameters were measured following the methodology of Nawaz, Xiong [14] using Minolta CR-300 Colorimeter (Osaka, Japan). These were measured by making a powder of each treatment by grinding and then packing into transparent polyethylene bags. Lightness (*L* *), redness (*a* *) and yellowness (*b* *) were calculated in triplicates for each treatment. Texture was calculated following the methodology of Nawaz, Xiong [20] using a three bending point (HDP/3PB) probe by means of TA-XT2 plus texture analyzer (stable micro system, Surrey, UK). The following settings were applied for measuring textural parameters (hardness and fructurability): load cell of 5 kg, test speed was 1 mm/s, compression was made up to 70% and an auto trigger type was used. The peak force (N) was measured immediately prior to breakage and fructurability was also calculated. For each treatment, six replicates from each cooking method were used and values were reported in means and standard deviation.

### 2.5. Pasting Properties

Pasting properties of fish meat snacks were measured by following the study of Yildiz, Yurt [21]. Regarding pasting properties, snacks were ground into powder form and used for pasting properties using Rapid Visco Analyzer (RVA)-4 series (Newport Scientific Pvt., Ltd., Narrabeen, NSW, Australia). Cooked snack powder (3 g) was added in 25 mL of distilled water, which was placed in RVA cylinder and stirred manually in order to make a slurry. This slurry was fixed in RVA equipment and stirred at 960 rpm for 10 s subsequently 160 rpm during analysis. After that, paste was kept at 50 °C for 60 s and then heated up to 95 °C in 3.7 min and hold at 95 °C for 2.5 min. Hereafter, temperature was dropped to 50 °C within 3.8 min and hold at 50 °C for 2 min. Meanwhile, peak, trough, breakdown, final and setback viscosities along with pasting temperature were measured. Among these, setback viscosities were calculated by subtracting the final viscosity from trough viscosity, whereas breakdown viscosities were measured by the difference between peak viscosity and trough viscosity. All calculations were done in triplicates for each treatment in order to calculate the mean values, which were further used for the statistical study.

### 2.6. Measurement of Protein Oxidation

Protein oxidation in terms of protein carbonyl contents was measured through following the methodology of Hu, Ren [6]. A spectrophotometer (Model F-4600, Hitachi, Tokyo, Japan) was used to measure the absorbance at 365 nm and carbonyl content was calculated using the absorption coefficient (22,000 (mol/L)^−1^ cm^−1^) and expressed as nmol/mg of protein.

### 2.7. Microstructure of Snacks

The microstructure of snacks was observed using a scanning electron microscope (NTC, JSM-6390LV; Tokyo, Japan) following the study of Nawaz, Xiong [17]. Briefly, cooked snacks (cross-sectional pieces) were freeze dried and then fixed into a bronze slide using double-edge sticky tape and then sputter-coated with platinum. The obtained images were observed at a magnification of 100 using an accelerated voltage of 15 kV.

### 2.8. Low-Field Nuclear Magnetic Resonance (LF-NMR) Analysis

Proton mobility distribution in cooked snacks was measured through low-field nuclear magnetic resonance using Minspec mq 20 low-field pulsed NMR spectrometer (Bruker, Ettlingen, Germany) following the previous methodology [22]. Briefly, transverse relaxation time (T2) was calculated at 20 MHz and at room temperature (25 °C) using a sequence based on Carr–Purcell–Meiboom–Gill (CPMG). The amount of proton in the population was proportional to the acquired peaks. Measurements were performed in triplicates for each treatment while the graph was plotted from the mean value of the triplicates.

### 2.9. Statistical Analysis

All analyses were performed in triplicates for obtaining the mean values and standard deviation. The obtained mean values were subjected for statistical analysis using one-way analysis of variance (ANOVA) and Duncan’s multiple range test using SPSS version 21.0 (SPSS statistics for windows, Armonk, IBM, Carp., New York, NY, USA). The statistically significant difference was set a level of *p* < 0.05. All graphs were drawn using origin pro 9 Software.

## 3. Results

### 3.1. Physiochemical Properties of Snacks

**Figure 1:**
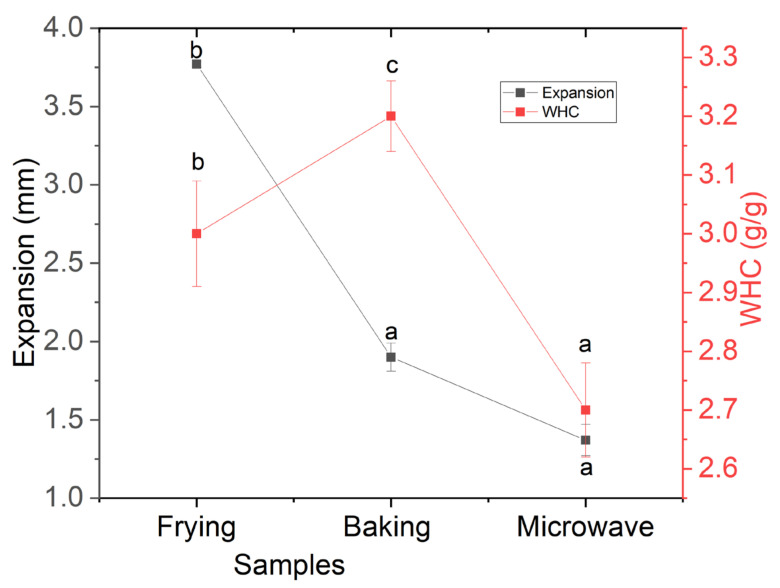
Effect of processing methods on expansion and WHC of fish meat-based snacks. The results of expansion and WHC are the mean and standard deviation of six and three snacks, respectively. Different letters (abc) over the error bars indicate the significant difference between processing methods at *p* < 0.05 using one-way ANOVA and Duncan’s multiple range test.

The expansion of snacks is an important parameter regarding consumer’s acceptability and appearance. Usually, expanded snacks foods are preferred, but more than 70% is unacceptable, which causes irregular volume and deshaped snacks [5]. The results of expansion are shown in Figure 1, and ranged from 1.25 to 3.75 mm, which revealed that there was a significant (*p* < 0.05) difference in frying and baking methods, while no statistically significant differences were observed between the baking and microwave methods. Although the values of expansion of baking and microwave were different, they were not statistically significant by means of the one-way ANOVA and DMR test. Overall expansion, which was almost 50% in all treatments, was attributed to the presence of starchy material and gluten protein, which both exhibit viscoelastic properties upon heating [14]. However, this expansion cannot be neglected owing to the addition of fish meat that has also been reported for the increased expansion in snack food. However, regarding the cooking methods, it was revealed that frying resulted in the highest expansion even it was more than 70% of the original volume. This is especially attributed to frying, in which high-temperature oil interacts with water, which raptures the surface and decreases the bulk density resulting in high expansion. Similar results about expansion were reported by in a previous study [20] when fish meat -based snacks were fried; however, the addition of >10% caused less expansion compared to control (devoid of meat). On the other hand, microwave heating is fast and uniform heating and based on the transition of glassy matrix to rubbery sate [23]. Moreover, during microwave heating, a sudden increase in temperature produces superheated steam and rapid escape of water molecules, which resulted in the transition to rubbery state of starch and started deformation and hindering of the proper volume. Therefore, the results of expansion in terms of baking and microwaving are acceptable in order to commercialize the product.

Water hydration capacity (WHC) predicts the behavior of processed foods in terms of extent of gelatinization and dextrinization, which are greatly affected by various thermal processing methods and types of ingredients within the matrix. The results shown in Figure 1 reveal that all treatments showed a significant (*p* < 0.05) difference and it was highest in baked snacks, while it was found to be lowest in the microwave followed by frying. The increased WHC in baking indicated the sufficient swelling of starch granules, which absorbed more water, while less WHC in the microwave might be due to the fact that during microwave heating, protein unfolds and expands more than starch, which wraps the starch granules, resulting in insufficient gelatinization. In case of frying, oil interacts with the matrix and creates hydrophobic conditions, which may result in less WHC. The improved values of WHC in case of baking method indicates the sufficient swelling of starch granules, which endorse the feasibility of the baking method compared to frying and the microwave.

The effect of processing method on color parameters is shown in Table 1. It was revealed that color parameters, especially lightness, were significantly (*p* < 0.05) increased in baking compared to microwave and frying treatments. Actually, the lower lightness in frying might be due to the oil, which led to the formation of crust and caused browning. Another reason could be the Maillard reaction between sugars and protein. Similar results were found by another study [24], which reported the level of lightness in various cooking methods as follows: baking > microwave > grilled > frying. Regarding redness, it was found that redness was significantly higher in those treatments having lower lightness as in the case of frying. Actually, frying takes places at higher temperature and oil makes the crust darker as compared to baking and microwave heating. On the other hand, microwave heating is a transfer of heat by means of radiation, which also prevents the excessive browning; however, the high temperatures caused by microwave heating resulted in low lightness compared to baking in our study, which was also evidenced by a previous study [25], in which yellowness was higher in frying followed by microwave and baking techniques. Our findings are in agreement with data reported by Cho, Lee [26] who showed that microwave cooking results in slightly lower lightness as compared to gas stove cooking of instant noodles. Since color is very important regarding sensory appearance, the findings regarding high lightness and low redness in the baking method have the potential to be considered for a good appearance.

### 3.2. Textural Properties of Snacks

**Figure 2:**
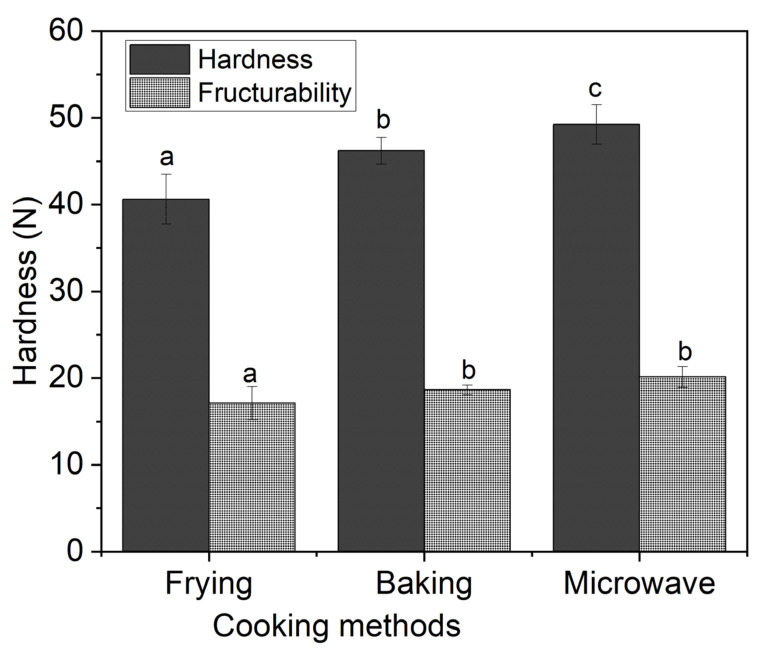
Effect of processing methods on textural characteristics of fish meat-based snacks. The results of texture parameters are the mean and standard deviation of six snacks for each treatment. Different letters (a,b,c) over the error bars indicate the significant difference between processing methods at *p* < 0.05 using one-way ANOVA and Duncan’s multiple range test.

Textural properties are of high interest regarding the consumer’s acceptability. Regarding snacks, hardness and fructurability are considered as important parameters. The results of textural properties are shown in Figure 2. It was disclosed that there was statistically significant (*p* < 0.05) difference in all treatments in terms of hardness, while it was highest in microwave cooking and lowest in frying treatments. On the other hand, regarding the results of fructurability, there was a significant (*p* < 0.05) difference between frying and baking or frying and microwave heating, but no difference was found in baking and microwave heating. Low hardness in frying could be attributed to that cooking method in which high-temperature oil created high expansion (more intercellular spaces) by rupturing the surface. In addition, as reported earlier [5], higher expansion causes lower hardness and decreased bulk density. The higher hardness in microwave treatment might be due to the rapid escape of water molecules due to the superheated steam production, which makes the compact structure of snacks. This was also evidenced in terms of low expansion for microwave-baked snacks. Another reason for the increased hardness in microwave heating might be due to the denaturation of myofibrillar proteins (actually actomyosin complex), which resulted in the shrinkage of muscle fibers, as compared to the modification of the connective tissues component during cooking. Due to rapid increase in temperature, water escaped from muscle fiber more quickly, which resulted in the less tender of meat as reported by a previous study [24]. Our results regarding the hardness of snacks are in agreement with the previous study, in which microwave heating resulted in the abnormal increase in the hardness of the meat compared to other cooking methods, especially grilling [24]. Low hardness results in soft matrix while high hardness makes meat difficult to chew; thus, the values for baking were within the range and can be considered as a reference for commercialization.

### 3.3. Pasting Properties of Snacks

**Figure 3:**
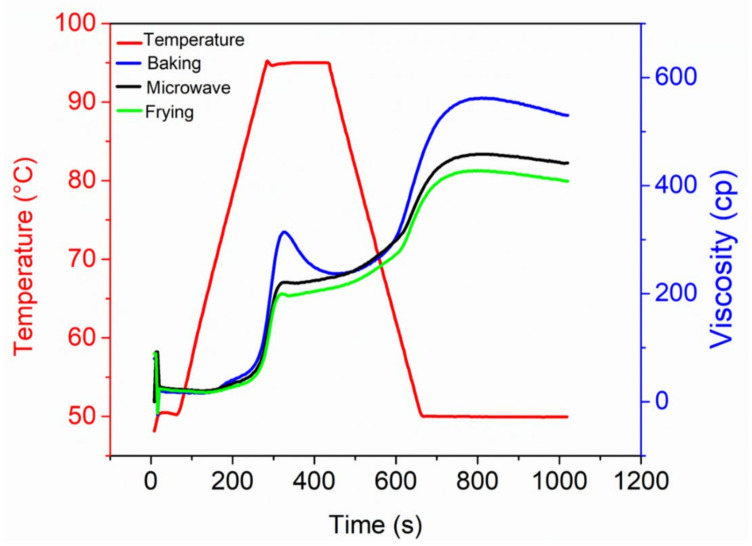
Pasting properties of fish meat-based snacks prepared by various processing methods.

Pasting properties are of extreme importance in order to predict the stability of products. These are usually determined by a combination of a complex process that occurs after the gelatinization of starch granules, which alter from swelling to rupture, amylose leaching and gel formation with the input of energy [21]. Thus, pasting properties greatly depend on processing methods along with the presence of other ingredients in a matrix, especially the type of starch [3], starch–protein interaction [5] and chemical reagents and additives [14]. The pasting properties of meat-based snacks prepared by various cooking methods are shown in Figure 3 and Table 2. Among these, peak viscosity, which is an indicative of highest viscosity acquired during gelatinization in the presence of water prior to its physical breakdown, was found to be significantly (*p* < 0.05) higher in baking, while it was lowest in frying. The increased peak viscosity in baking could be due to long time and gradual heating of starch granules that attained sufficient swelling time. On the other hand, the rapid heating or instant heating in microwave heating may partially inhibit the starch gelatinization or be due to the proteins, which showed intensive viscoelastic properties upon microwave heating. The decreased peak viscosity in frying could be due to the oil droplets that behaved as barrier and prevented the swelling of starch granules. The increased in pasting properties of baked snacks could be due to the proper gelatinization of starch, which was inhibited in microwave cooking due to sudden increase in temperature. Thus, the starch granules sufficiently swelled in baking, and pasting properties were increased.

Tough viscosity signifies the ability of paste to withstand breakdown after gelatinization when subjected to cooling and was found to be higher again in baking followed by microwave and frying treatments. This indicates that snacks cooked by baking and microwave heating were able to form a stable paste compared to frying, which was also evidenced by the results of WHC. Similarly, the results of final viscosity are also in line with peak and final viscosity, indicating the importance of cooking methods especially for baking, in which a slow heating rate provided ample time for the gradual swelling of starch granules, which were easily disrupted by sharing action of paddle [27]. The reason for the low pasting properties in microwave heating might be due to the instant heating of microwave, which degraded the starch granules leading to the disruption of hydrogen bonds and glycosidic linkage, resulting in the weakening of the granules’ integrity [28]. Breakdown values represent the strength of granules, whereas setback values suggest the degree of rearrangement of starch molecules after gelatinization and determine the transformation of viscous liquid to gel. From the results, it was obvious that final and setback viscosities were significantly higher in baking, which again endorses the slow heating for a proper starch–protein network development. On the other hand, there was no significant difference in the values of setback viscosities among frying and microwave heating. Therefore, higher values for pasting properties especially in baking and microwave cooking indicate the stability of snack food.

### 3.4. Protein Carbonyl Content in Cooked Snacks

**Figure 4:**
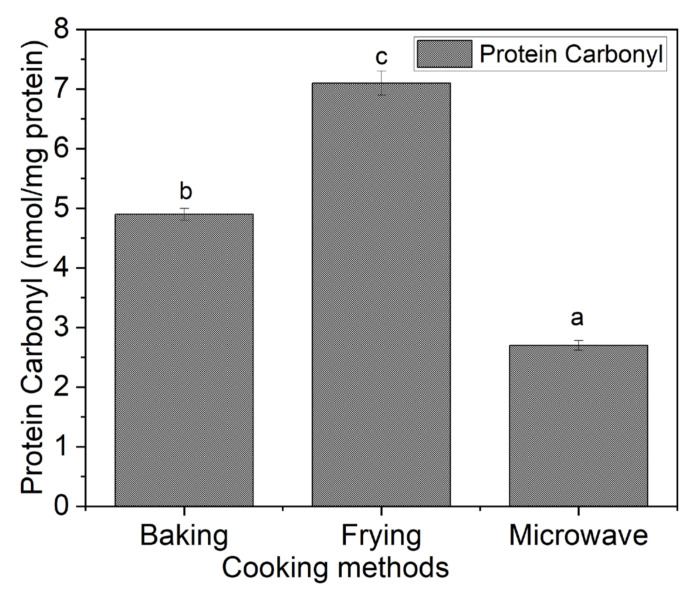
Protein carbonyl content of fish meat-based snacks prepared by different cooking methods. Reported values are mean of triplicate of each treatment and small letter over bar represent significant difference using one-way ANOVA and DMR’s test at a significance level of *p* < 0.05.

Protein oxidation has remained the critical challenge for meat industry, which is initiated by various factors, such as types and composition of meat, processing methods, intensity of cooking temperature and time and the presence of non-proteinous material. The results of protein carbonyl content are shown in Figure 4. The results revealed that protein carbonyl content, an important biomarker for measuring protein oxidation, was found to be significantly (*p* < 0.05) highest in frying followed by baking and microwave oven heating. The highest oxidation induced by frying could be due the lipids that are also a leading cause of protein oxidation as reported by previous study [13]. Generally, during heat treatment, meat loses its oxidative potential and cellular damage of cells occurs, which makes it more expose to oxygen, subsequently trigging the reactive oxygen species (ROS) that attack the lipid and protein molecules. Moreover, cooking promotes the oxidative cleavage of porphyrin ring, releasing the heme iron (a pro-oxidant), which accelerate oxidative deterioration [6,29].

The increased in protein oxidation in frying could be attributed to oil absorption in fillets, which exposed the proteins to oxidative cleavage. However, the increased protein oxidation in baking after frying could be attributed to long cooking time along with higher temperature, which are responsible for protein oxidation, as reported previously [6]. The increased oxidation by frying has also been reported by previous studies [9]. On the other hand, the lowest protein carbonyl content in microwave heating endorses the suitability of microwave heating, which was for less time, in terms of safety point of view.

### 3.5. Microstructure of Snacks

**Figure 5:**
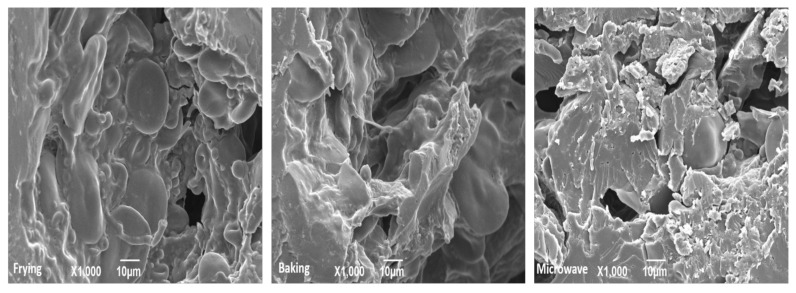
Microstructural analysis of fish meat-based snacks prepared by various processing methods.

The micrographs of snacks made from different cooking methods are shown in Figure 5. The observation revealed that frying method had void holes and an irregular matrix, which was also evidenced in the form of higher expansion. Some starch granules were of small size indicating the insufficient swelling of granules, which resulted in decreased pasting properties of that method. Regarding baking method, a compact structure was found with swelled and gelatinized starch granules indicating a uniform matrix. Moreover, the starch granules were embedded within the protein matrix, suggesting a uniform starch–protein network. The spaces between starch and protein were less visible. On the other hand, the image for microwave baking was quite different from those of frying and baking with an uneven structure and non-uniform matrix. Irregular blocks were visible, which may indicate the weak interaction between starch and protein. However, the morphology was much better than frying in terms of irregular spaces and starch granules. The photos presented in Figure 5 are in accordance with previous study with same structure [12].

### 3.6. LF-NMR Analysis of Snacks

**Figure 6:**
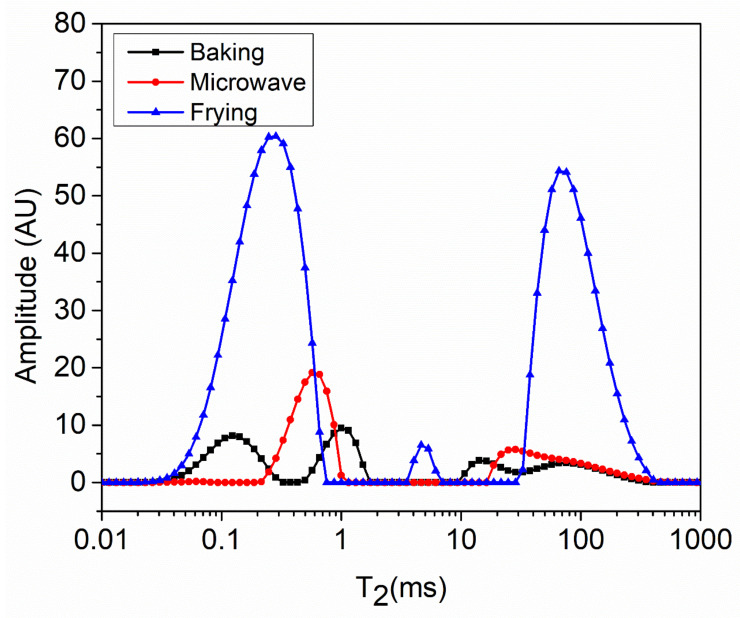
LF-NMR analysis of fish meat-based snacks prepared by various processing methods.

The changes in water distribution and state of water during the thermal processing are essential parameters that predict the stability, physiochemical changes and shelf-life of a product. Water distribution or water mobility within the cooked matrix are usually determined by the relaxation time (T_2_) by means of LF-NMR analysis [22]. The T_2_ relaxation time is further categorized into three subsections: T_21_ ranges from 0.1 to 1 ms and indicates the tightly bound water, T_22_ (1–100 ms) signifies the immobilized water, whereas T_23_ represents the unbound water [14]. Generally, shorter relaxation time and shorter amplitude disclose a strong association between solids and water within a matrix, whereas long amplitude shows weak interaction of water in a heterogenous system. The results of LF-NNMR analysis are shown in Figure 6. The findings regarding T_21_ showed that relaxation time and amplitude both were highest in frying, where they were lowest in baking followed by microwave heating, suggesting that more tightly bound water was present in baked snacks compared to frying. Again, the peak for the amplitude of immobilized water T_22_ (1–100 ms) was highest in frying, which indicates the less-immobilized water in fried snacks compared to microwave and baking treatments. It was noteworthy to observe that peak for microwave cooking was very low, which suggest the strong interaction of water with other ingredients, and this indicates the proper starch–protein network development in microwave and baking methods. A similar trend was observed for T_23_ relaxation time in which the frying method showed the highest amount of unbound water. Kexin, Xu [22] also found that the frying process of turbot flesh resulted in high amount of unbound water as compared to stewing. This could be due to the oil that created a hydrophobic environment within the matrix resulting in insufficient swelling and gelatinization, which was also evidenced in pasting properties. On the other hand, the amplitude peaks for baking and microwave were almost similar in this region, which suggests that snacks prepared with these methods revealed the least amount of unbound water. This suggests that water was tightly bound within quaternary and tertiary structures of proteins during thermal processing. The high amount of unbound water causes loosening of the structure and texture, and it was leached out during heating and especially in frying methods. This unbound water was retained in the matrix owing to the crust formation by the frying method and was observed in the form of unbound water. In addition, with slow cooking for a long time, such as in baking, the starch granules absorbed sufficient amount of water with the full capacity of gelatinization, resulting in the tightly bound water within the matrix [5].

The role of water is of high importance during thermal processing, as water acts a plasticizer and promotes interaction between the molecules of starch and protein. In addition, the role of the cooking medium is very important. Once possibility in fried snacks (less immobilized and tight water) is that the frying method might breakdown the fiber bundles’ network, resulting in the leaching of free water and less-immobilized water. The results are in accordance with the study of Liu, Dong [13]. Moreover, frying caused more protein aggregation and confirmation, which alter the tissue gap in meat muscle, leading to availability of free water, which was also evidenced in the form of higher protein carbonyl content in frying [6]. Similar results were reported by another study [12] in which the microwave method resulted in a higher amount of immobilized water compared to other cooking methods.

## 4. Conclusions

The present study reported three most used methods for fish meat-based snacks and evaluated quality, safety and structural parameters. Due to high oil uptake in frying, which causes serious diseases such as cardiovascular diseases, consumer’s choice is moving toward non-oil-based methods, such as baking and microwave heating. The major findings revealed that baking and microwave cooking could be the alternative choices instead of frying, which not only improved the quality (color and texture), but also enhanced the physiochemical (WHC, pasting properties), safety (protein oxidation) and water distribution properties. The baking can be considered as an optimum method for meat-based snacks, especially for proper starch–protein interaction, which improved texture, WHC and pasting properties. The microwave method can be considered as the best choice in terms of decreased protein oxidation and water mobility, especially the presence of tightly and immobilized water, which all highly endorse the appropriateness of microwave cooking. However, in case of microwave cooking, as protein behaved extensively in response of microwaves, there is a need to further investigate the mechanism and optimum conditions for meat-based snacks. Beside this, there is need to conduct storage stability of fish meat-based snacks for these cooking methods. With all these investigations, the appropriateness of processing methods can be enhanced and commercialized for fish meat-based snacks.

## Figures and Tables

**Table 1:** Physiochemical properties of fish meat-based snacks prepared by various processing methods.

Samples	Frying	Baking	Microwave
Lightness (L *)	79.40 ± 0.50 ^a^	85.69 ± 0.43 ^c^	83.40 ± 0.32 ^b^
Redness (a *)	5.93 ± 0.17 ^c^	3.32 ± 0.21 ^a^	3.97 ± 0.25 ^b^
Yellowness (b *)	17.38 ± 0.16 ^b^	13.85 ± 0.34 ^a^	16.83 ± 0.19 ^a,b^

The results of color and texture parameters are the mean and standard deviation of three and six snacks for each treatment, respectively. Different letters (a,b,c) in columns indicate the significant difference between processing methods at *p* < 0.05 using one-way ANOVA and Duncan’s multiple range test.

**Table 2:** Pasting properties of fish meat-based snacks prepared by various processing methods.

Samples	Peak Viscosity (RVU)	Trough Viscosity (RVU)	Breakdown Viscosity (RVU)	Final Viscosity (RVU)	Setback (RVU)	Temperature (°C)
Baking	328 ± 30.50 ^b^	245 ± 20.50 ^b^	83 ± 22.50 ^b^	549 ± 30.22 ^c^	304 ± 25.50 ^b^	95.25 ± 0.09 ^a,b^
Microwave	237 ± 10.61 ^a,b^	221 ± 0.50 ^a^	16 ± 9.50 ^a^	442 ± 14.50 ^b^	221 ± 09.69 ^a^	95.00 ± 0.04 ^b^
Frying	217 ± 12.80 ^a^	200 ± 0.50 ^a^	17 ± 5.50 ^a^	408 ± 14.50 ^a^	208 ± 10.22 ^a^	94.65 ± 0.50 ^a^

The results of pasting properties are the mean and standard deviation of triplicates of each treatment, respectively. Different letters (a,b,c) in rows indicate the significant difference between processing methods at *p* < 0.05 using one-way ANOVA and Duncan’s multiple range test.

## Data Availability

Data are openly available for all readers after publication.
